# Associations of serum and dialysate electrolytes with QT interval and prolongation in incident hemodialysis: the Predictors of Arrhythmic and Cardiovascular Risk in End-Stage Renal Disease (PACE) study

**DOI:** 10.1186/s12882-019-1282-5

**Published:** 2019-04-18

**Authors:** Esther D. Kim, Jacqueline Watt, Larisa G. Tereshchenko, Bernard G. Jaar, Stephen M. Sozio, W. H. Linda Kao, Michelle M. Estrella, Rulan S. Parekh

**Affiliations:** 1 Department of Epidemiology, Johns Hopkins Bloomberg School of Public Health, Baltimore, MD USA; 2 Welch Center for Prevention, Epidemiology, and Clinical Research, Baltimore, MD USA; 3 Child Health Evaluative Sciences, The Hospital for Sick Children, Hospital for Sick Children, 555 University Ave, Toronto, ON M5G 1X8 Canada; 4 Knight Cardiovascular Institute, Oregon Health and Science University, Portland, OR USA; 5 Department of Medicine, Johns Hopkins University School of Medicine, Baltimore, MD USA; 6 Nephrology Center of Maryland, Baltimore, MD USA; 7 Department of Pediatrics and Medicine, The Hospital for Sick Children, University Health Network and University of Toronto, 555 University Ave, Toronto, ON Canada; 8 Johns Hopkins University, School of Public Health, Baltimore, Maryland, USA

**Keywords:** QT interval, QT prolongation, Arrhythmia, Electrolytes, Hemodialysis

## Abstract

**Background:**

Prolonged QT interval in hemodialysis patients may be associated with sudden cardiac death, however, few studies examined the longitudinal associations of modifiable factors such as serum and dialysate concentrations of calcium, potassium, and magnesium with corrected QT (QTc) prolongation in incident hemodialysis patients.

**Methods:**

In 330 in-center hemodialysis participants from the PACE study who were followed up for one year, we examined the associations of predialysis serum electrolytes (total calcium [Ca], corrected Ca [cCa], ionized Ca [iCa], potassium [K], magnesium [Mg]), dialysate (dCa and dK), and serum-to-dialysate gradient measures with QTc interval and prolongation (≥460 ms in women and ≥ 450 ms in men).

**Results:**

At the first study visit, 47% had QTc prolongation. Lower iCa and K were associated with longer QTc interval independent of potential confounders (QTc difference = 8.55[95% CI: 2.13, 14.97] ms for iCa; QTc difference = 9.89[1.58, 18.20] ms for K). Lower iCa was also associated with a higher risk of QTc prolongation. At 1 year of follow-up, 31% had persistent QTc prolongation. In longitudinal analyses, the associations of iCa and K with QTc interval remained significant, and lower K was associated with a higher risk of QTc prolongation while the association of iCa with QTc prolongation was borderline statistically significant. Serum Mg, dCa or dK, and respective gradients were not associated with QTc interval or prolongation.

**Conclusion:**

Prolonged QTc is very common in incident hemodialysis participants and persists over follow-up. Ionized Ca and K are consistently inversely associated with QTc prolongation, which suggests closer monitoring for a low calcium or potassium level to mitigate risk.

**Electronic supplementary material:**

The online version of this article (10.1186/s12882-019-1282-5) contains supplementary material, which is available to authorized users.

## Background

Sudden cardiac death (SCD) is the leading cause of death in hemodialysis. Coronary events and atherosclerotic risk factors are only weakly associated with SCD in hemodialysis, which suggests that lethal ventricular arrhythmias in this population may largely contribute to high rates of SCD [1, 2]. On hemodialysis, arrhythmias can occur during and shortly after receiving treatment, independent of traditional cardiovascular risk factors [3, 4]. Despite the increased risk of SCD, there is no consensus on dialysis-related risk factors for the development of arrhythmias.

Calcium, potassium, and magnesium may contribute to the development of arrhythmias during hemodialysis as these cations play a major role in development of the ventricular action potential and propagation of the electrical impulse [4–6]. During hemodialysis, a rapid decrease in serum calcium or potassium can result in prolonged QT interval and increased QT dispersion [6–9]; however, findings from observational studies and randomized control trials in hemodialysis are inconsistent. In hemodialysis, serum calcium levels fluctuate due to routine intermittent nature of the treatments and certain medications. The optimal serum calcium range, and type of calcium measurement remains unclear as guidelines are targeted to lower risk of vascular calcification [10].

Observational studies of prevalent dialysis patients demonstrated associations of calcium and potassium concentrations with the risk of arrhythmias and arrhythmic events. For example, low dialysate calcium concentration was associated with longer corrected QT (QTc) interval (> 440 ms); larger serum-to-dialysate calcium gradient was associated with a higher risk of sudden cardiac arrest [4, 11]; and low dialysate potassium concentration and low and high predialysis potassium levels were associated with an increased risk of sudden cardiac arrest [12]. Conversely, findings from a trial of 3883 prevalent hemodialysis participants report that baseline dialysate calcium and serum-to-dialysate calcium gradient are not associated with cardiovascular outcomes including arrhythmic events [13]. While many of these studies examined the relationship of calcium and potassium with arrhythmic events – often cross-sectionally, fewer studies have examined repeated measures of arrhythmic risk and compared various measures of longitudinal serum and dialysate electrolytes to the development of arrhythmias such as QTc in incident hemodialysis patients. The objective of this study was to examine the cross-sectional and longitudinal associations of serum and dialysate electrolytes with QTc interval and prolongation in incident hemodialysis patients.

## Methods

### Study design and population

This study utilized data from the Predictors of Arrhythmic and Cardiovascular Risk in End Stage Renal Disease (PACE) study. Details of the PACE protocol were described previously [14]. Briefly, PACE is a prospective cohort of incident hemodialysis patients enrolled from 27 outpatient dialysis units in the greater Baltimore area. Eligible participants were adults (> 18 years of age) who were within 6 months of hemodialysis initiation (*n* = 568). Participants were excluded if they had a pacemaker or an automatic implantable cardiac defibrillator. Participants who completed a baseline study cardiovascular clinic visit (*n* = 397) were eligible for the baseline analysis and were further excluded from analyses if they had detectable atrial fibrillation by electrocardiogram (ECG) during the study clinic visits (*n* = 6) or if the ECG assessments were either incomplete or uninterpretable (*n* = 24). We further excluded participants who were missing any of the serum or dialysate electrolyte measurements (*n* = 33). Participants who completed both baseline and a 1 year follow-up Institute for Clinical and Translational Research visits were included in the longitudinal analysis.

The study protocol was approved by the institutional review board of the Johns Hopkins School of Medicine, MedStar Health Systems, and by the medical director of each dialysis unit. All participants provided written informed consent.

### Data collection

Participants completed standardized self-report questionnaires in the dialysis unit and at the study visit at baseline, and at 1 year. Cardiac evaluations were conducted on a non-dialysis day and included ECG, echocardiogram, and blood pressure assessments by trained technologists or study staff. Dialysis treatments and laboratory data were provided by DaVita Clinical Research and MedStar Health Systems.

Exposure variables of interest were electrolyte levels: serum calcium, potassium, and magnesium, dialysate concentrations of calcium and potassium, and corresponding serum-to-dialysate gradients. Serum calcium, albumin-corrected calcium, and serum potassium were assessed as measurements closest to the study clinic visit. Serum ionized calcium and serum magnesium, as well as the serum pH level, were assessed from blood samples collected at the study visits on a non-dialysis day. Dialysate calcium and potassium concentration values closest to the study visit were collected. Serum-to-dialysate gradient was calculated for each calcium or potassium measurement and defined as the difference between serum calcium (total or corrected) and dialysate calcium level, or as the difference between serum potassium and dialysate potassium level after converting all units to mEq/l [11]. All electrolyte measures were collected at study baseline and at 1 year.

Outcome of interest was QTc interval and QTc prolongation. QT interval was measured as an average on the 5-min ECG (Norav Medical Ltd., Thornhill, On, Canada) and corrected using Hodges formula, as the Bazett formula was shown to overestimate the prevalence of QT prolongation [15]. QTc prolongation was defined as QTc interval ≥ 450 ms in males and ≥ 460 ms in females [16]. QTc interval and prolongation were measured at study baseline and at 1 year of follow-up.

Additional demographic variables included baseline age, sex, and self-reported ethnicity. Body mass index was assessed at the baseline clinic visit. Medical records for the 6 months prior to initiation of dialysis were reviewed by an adjudication committee of physicians to derive the Charlson comorbidity index. Antihypertensive medications, cinacalcet, and QT prolonging medications (Additional file 1: Table S1) were recorded during study visits [17]. Resting blood pressure measure was the average of three blood pressure measures in a seated position after 5 min of rest. Baseline left ventricular mass was measured by echocardiogram and left ventricular mass index (LVMI) was calculated using Devereux’s formula [18]. Predialysis hemoglobin was examined as a three-month average prior to the study clinic visit.

### Statistical analysis

All normally distributed continuous variables were examined using means (± standard deviation [SD]) and compared across groups using the Student t-test. Median (interquartile range [IQR]) and the Wilcoxon rank sum test were used for non-normally distributed continuous variables. Categorical variables were examined using frequencies (%) and compared using the chi-square test or Fisher’s exact test.

Multiple analyses were performed to examine the associations of serum electrolytes (calcium, potassium, and magnesium) with QTc interval and prolongation. Baseline association of each serum electrolyte measure with QTc interval was examined separately using univariable and multivariable linear regression. Baseline associations of serum electrolyte measures with continuous QTc prolongation were assessed using Poisson regression with a robust error variance [19]. Longitudinal association between each exposure and QTc interval was assessed using the generalized estimating equations (GEE) approach with a Gaussian distribution. Similarly, longitudinal associations between each exposure and QTc prolongation was modeled using GEE with a log link and Poisson distribution. For longitudinal models, we estimated robust variance, assumed an exchangeable within-person correlation structure, and included time-updated values for exposures, outcomes, and covariates that were also measured at 1 year of follow-up. We also compared the relative strengths of the associations of calcium, potassium, and magnesium measures with QTc interval and prolongation by simultaneously adjusting for each serum calcium, potassium, or magnesium measure in the same model using standardized coefficients presented as a change in QTc interval per 1 standard deviation change in the exposure variable. The multivariable models included age, sex, ethnicity, Charlson comorbidity index, non-dialysis systolic blood pressure, left ventricular mass index, and use of beta-blocker, renin-angiotensin-aldosterone system blockade (RAAS), cinacalcet, QT-prolonging medications (Additional file 1: Table S1), and serum pH (in models with ionized calcium and serum magnesium), and this was determined using previously reported associations with arrhythmia and a forward model building approach that involved evaluating changes in effect-size. Potential non-linear relationships of continuous exposures and outcomes were examined using restricted cubic splines.

We performed several sensitivity analyses to test the robustness of our findings at baseline by follow-up status. We compared the baseline characteristics between participants who completed both the first study visit and at 1-year follow-up and participants who were later lost to follow-up. We also repeated the baseline analyses after excluding participants who were lost to follow-up at 1 year. Missing values were imputed using the multiple imputation by chained equation method [18]. Imputed variables were LVMI (2% missing), beta-blocker (10%), RAAS (10%), cinacalcet (10%), and non-dialysis systolic blood pressure (1%). For all analyses, a two-tailed *p* value of < 0.05 was considered significant. All analyses were performed using Stata 14 (College Station, Texas).

## Results

### Study population

The study population for the baseline cross-sectional analysis included 330 participants. Median time on dialysis was 104 (interquartile range [IQR]: 78, 147) days at their first study visit. The mean age was 54.6 ± 13.3 (standard deviation [SD]) years and the majority were male (62.4%), African-American (73.6%), diabetic (56.7%), and received beta-blocker medication (69.5%) (Table 1). Mean total calcium, corrected calcium, and total potassium serum levels were 8.8 ± 0.6 mg/dl, 9.0 ± 0.6 mg/dl, and 4.4 ± 0.6 mEq/l, respectively. The mean ionized calcium and magnesium serum levels were 1.15 ± 0.07 mmol/l and 1.76 ± 0.24 mEq/l, respectively. At baseline, 155 (47.0%) participants had evidence of QTc prolongation.

**Table 1:** Baseline demographic factors, cardiovascular disease risk factors, and laboratory measurements in 330 participants

Variables	Mean (± SD), median (IQR), frequency (%)
Demographic factors	
Age, years	54.6 (± 13.3)
Sex	
Male	206 (62.4)
Female	124 (37.6)
Ethnicity	
Non-African American	87 (26.4)
African American	243 (73.6)
Physical information	
Body mass index, kg/m^2^	27.8 (23.5, 33.3)
Non-dialysis study visit systolic, mmHg	137.8 (± 25.3)
Non-dialysis study visit diastolic, mmHg	75.0 (± 14.7)
Non-dialysis study visit pulse pressure, mmHg	62.8 (± 18.1)
Comorbidities	
Hypertension	330 (100.0)
Hypercholesterolemia	228 (69.1)
Diabetes	187 (56.7)
Coronary artery disease	116 (35.2)
Charlson comorbidity index	5 (4, 6)
Medications	
Total number of antihypertensive medications	2.7 (± 1.3)
Beta-blocker	207 (69.5)
RAAS^a^ blockade	127 (42.6)
Calcium channel blockers	183 (61.4)
Alpha blockers	32 (10.7)
Vasodilators	102 (34.2)
Diuretic	68 (22.8)
Cinacalcet	15 (5.0)
QT prolonging medication^b^	118 (35.8)
Cardiac parameters	
Left ventricular mass index, g/m^2.7^	61.3 (50.1, 80.4)
Left ventricular ejection fraction, %	65.5 (± 11.9)
Laboratory measurements	
3-month average hemoglobin, g/dl	10.8 (± 1.2)
pH	7.34 (± 0.04)
Laboratory measurements	
Total calcium^c^, mg/dl	8.8 (± 0.6)
Corrected calcium^c^, mg/dl	9.0 (± 0.6)
Ionized calcium^d^, mmol/l	1.15 (± 0.07)
Total potassium^c^, mEq/l	4.4 (± 0.6)
Magnesium^d^, mEq/l	1.76 (± 0.24)
Dialysate measurements	
Calcium^c^, mEq/l	
2	139 (42.1)
2.25	14 (4.2)
2.5	174 (52.7)
3	3 (0.9)
Potassium^c^, mEq/l	
1	5 (1.5)
2	278 (84.2)
3	47 (14.2)
Serum-dialysate measurements	
Total calcium^c^, mEq/l	2.1 (± 0.4)
Corrected calcium^c^, mEq/l	2.2 (± 0.4)
ECG measurements	
QT interval^d^, ms	436.0 (± 55.6)
Corrected QT interval^d^, ms	455.4 (± 46.0)
QT prolongation^d^	155 (47.0)

^a^Renin-angiotensin-aldosterone system blockade includes angiotensin-converting-enzyme inhibitor and angiotensin II receptor blocker

^b^Listed in Additional file 1: Table S1

^c^Measurement closest to the study clinic visit

^d^Non-dialysis (interdialytic) measurements

### Serum and dialysate electrolytes with prolonged QTc at baseline

**Fig. 1:**
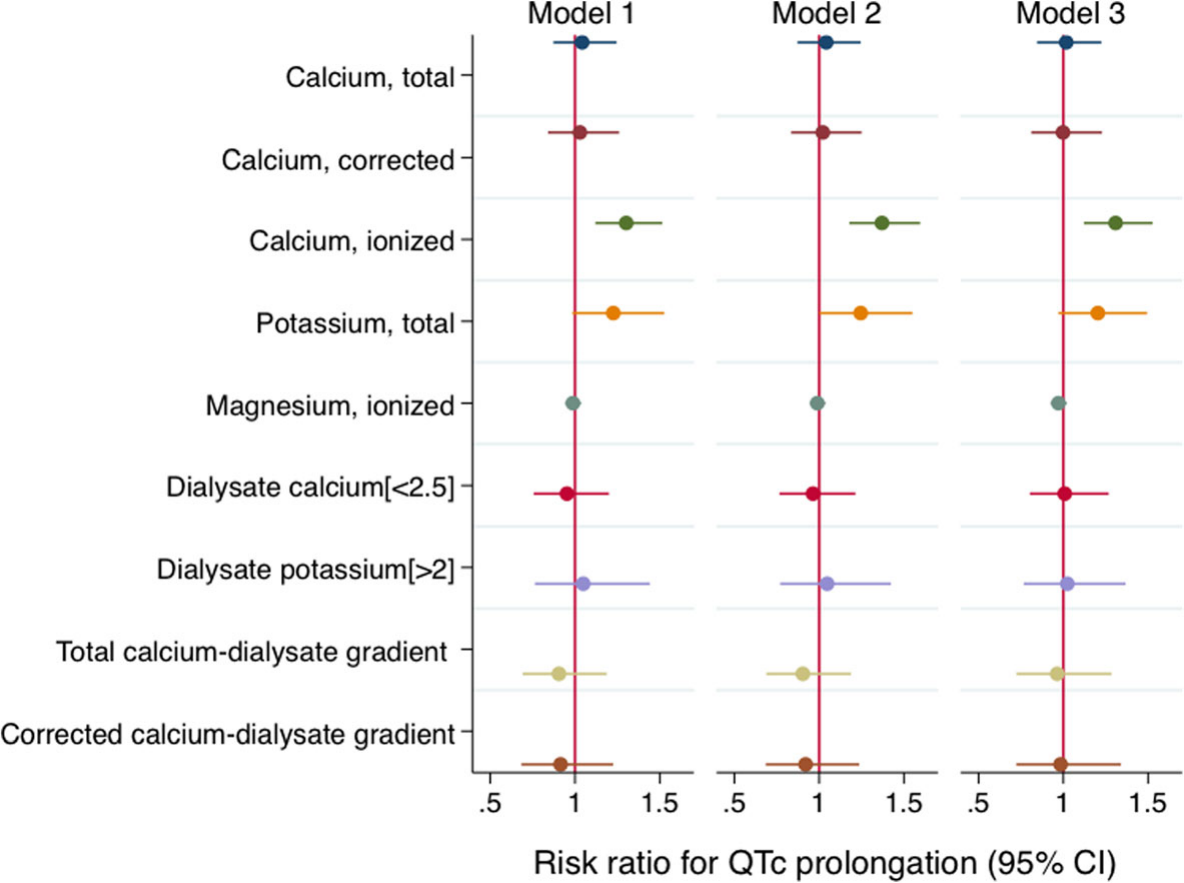
Associations of serum, dialysate, and serum-dialysate gradient measures with the risk of QTc prolongation at baseline in 330 incident dialysis participants.* Total calcium, per 1 mg/dl decrease; corrected calcium, per 1 mg/dl decrease; ionized calcium, per 0.1 mmol/l decrease; potassium, per 1 mEq/l decrease; magnesium, per 0.1 mEq/l decrease; dialysate calcium [< 2.5] = dialysate calcium < 2.5 mEq/l in reference to 2.5 mEq/l; dialysate potassium [> 2] = dialysate potassium > 2 mEq/l in reference to 2 mEq/l; total calcium-dialysate gradient, per 1 mEq/l increase in difference; corrected calcium-dialysate gradient, per 1 mEq/l increase in difference. Model 1 includes the main exposure (one of serum, dialysate, or gradient measurements). Model 2 includes model 1, age, sex, and ethnicity. Model 3 includes model 2, Charlson comorbidity index, non-dialysis systolic blood pressure, left ventricular mass index, and use of antihypertensive medication, renin-angiotensin-aldosterone system blockade, cinacalcet, and QT-prolonging medication. Models with ionized calcium and magnesium also include serum pH

At baseline, each 0.1 mmol/l decrease in ionized calcium was significantly associated with 8.55 (95% CI: 2.13, 14.97) ms longer QTc interval, even after adjusting for demographic factors, Charlson comorbidity index, non-dialysis systolic blood pressure, left ventricular mass index, and use of antihypertensive medications, cinacalcet, and QT-prolonging medications (Table 2). Each 1 mEq/l decrease in serum potassium was also associated with longer QTc interval after adjusting for potential confounders. Lower ionized calcium level was also consistently associated with a higher risk of QTc prolongation (Fig. 1, Additional file 1: Table S2). Lower potassium also associated with a higher risk of QTc prolongation but this was attenuated slightly after adjustment for potential confounders. Other measures of calcium, magnesium, dialysate measures, and serum-to-dialysate gradient measures did not significantly associate with QTc measures.

**Table 2:** Baseline associations of serum and dialysate electrolytes with QTc interval in 330 incident dialysis patients

Variables	Model 1		Model 2		Model 3	
	QTc difference (95% CI), ms	P	QTc difference (95% CI), ms	P	QTc difference (95% CI), ms	P
Serum measurements						
Total calcium, per 1 mg/dl decrease	+ 3.23 (−4.44, 10.89)	0.41	+ 4.46 (− 2.84, 11.75)	0.23	+ 2.70 (− 4.67, 10.06)	0.47
Corrected calcium, per 1 mg/dl decrease	+ 2.27 (−6.48, 11.03)	0.61	+ 3.46 (− 4.89, 11.80)	0.42	+ 1.56 (− 6.65, 9.77)	0.71
Ionized calcium^a^, per 0.1 mmol/l decrease	+ 9.77 (2.97, 16.57)	0.01	+ 10.32 (3.87, 16.78)	0.002	+ 8.55 (2.13, 14.97)	0.01
Potassium, per 1 mEq/l decrease	+ 11.08 (2.18, 19.98)	0.02	+ 10.97 (2.50, 19.43)	0.01	+ 9.89 (1.58, 18.20)	0.02
Magnesium^a^, per 0.1 mEq/l decrease	+ 0.34 (− 1.73, 2.40)	0.75	+ 0.35 (− 1.61, 2.31)	0.73	− 0.21 (− 2.13, 1.72)	0.83
Dialysate measurements						
Calcium						
2.5 mEq/l	reference		reference		reference	
< 2.5 mEq/l	+ 1.27 (− 8.70, 11.24)	0.80	− 0.90 (− 10.55, 8.74)	0.86	+ 0.95 (− 8.52, 10.42)	0.84
Potassium						
2 mEq/l	reference		reference		reference	
> 2 mEq/l	−2.73 (− 16.96, 11.49)	0.71	−3.53 (− 17.01, 9.94)	0.61	−3.98 (− 17.16, 9.21)	0.56
Serum-to-dialysate gradients						
Total calcium, per 1 mEq/l increase in difference	−3.33 (− 15.22, 8.55)	0.58	−6.84 (− 18.30, 4.62)	0.24	− 3.04 (− 14.57, 8.49)	0.61
Corrected calcium, per 1 mEq/l increase in difference	− 1.81 (− 14.66, 11.03)	0.78	− 5.40 (− 17.82, 7.02)	0.40	− 1.44 (− 13.72, 10.83)	0.82

Model 1 includes the main exposure (one of serum, dialysate, or gradient measurements)

Model 2 includes model 1, age, sex, and ethnicity

Model 3 includes model 2, Charlson comorbidity index, non-dialysis systolic blood pressure, left ventricular mass index, and use of antihypertensive medication, renin-angiotensin-aldosterone system blockade, cinacalcet, and QT-prolonging medication

^a^Models with ionized calcium and magnesium also include serum pH

### Comparison of participants with and without follow-up

A total of 146 participants completed the subsequent follow up visit a year later for the longitudinal analysis. Participants were not included in the longitudinal analytical sample due to death (*n* = 35), kidney transplant or transfer to peritoneal dialysis (*n* = 24), moved out of the area (*n* = 28), withdrawal from study or reached end of study (*n* = 20), non-adherence or missing follow-up visit (*n* = 77). Comparison of the baseline ECG measures by mortality status at 1 year demonstrated no significant differences (QTc interval, *P* = 0.5; QTc prolongation, *P* = 0.6). Among participants who survived to 1 year after their first study visit, those who completed the follow-up visit had higher proportion of African Americans; however, other characteristics did not differ significantly (Additional file 1: Table S3).

### Serum and dialysate electrolytes with prolonged QTc over 1 year of follow-up

At 1 year, 45 of 146 (30.8%) participants had QTc prolongation, of whom, 32 (73.3%) had persistent QTc prolongation (defined as having QTc prolongation at both time points). Over 1 year of follow-up, QTc interval decreased by 15.16 ms (95% CI: -21.80, − 8.53); total serum and ionized calcium did not change significantly (change of 0.03 [95% CI: -0.10, 0.15] mg/dl for total serum calcium; change of 0.02 [95% CI: -0.12, 0.16] mmol/l for ionized calcium); and serum potassium increased by 0.18 (95% CI: 0.07, 0.29) mEq/L. Distributions of dialysate calcium and potassium concentrations remained similar.

Longitudinal analysis demonstrated that lower ionized calcium and serum potassium remained significantly associated with a longer QTc interval over time in univariable and multivariable analysis (Table 3). Lower total calcium levels were also associated with longer QTc interval but attenuated slightly after adjusting for potential confounders.

**Table 3:** Longitudinal associations of serum and dialysate electrolytes with QTc interval in 146 incident dialysis patients

Variables	Model 1		Model 2		Model 3	
	QTc difference (95% CI), ms	P	QTc difference (95% CI), ms	P	QTc difference (95% CI), ms	P
Serum measurements						
Total calcium, per 1 mg/dl decrease	+ 9.13 (0.65, 17.61)	0.04	+ 8.89 (1.20, 16.58)	0.02	+ 7.06 (− 0.59, 14.72)	0.07
Corrected calcium, per 1 mg/dl decrease	+ 8.29 (−1.29, 17.88)	0.09	+ 7.71 (− 0.91, 16.34)	0.08	+ 5.74 (− 2.78, 14.26)	0.19
Ionized calcium^a^, per 0.1 mmol/l decrease	+ 9.79 (3.17, 16.41)	0.004	+ 10.18 (4.02, 16.33)	0.001	+ 9.05 (3.28, 14.83)	0.002
Potassium, per 1 mEq/l decrease	+ 9.08 (2.23, 15.94)	0.01	+ 9.87 (3.12, 16.62)	0.004	+ 9.43 (2.89, 15.98)	0.01
Magnesium^a^, per 0.1 mEq/l decrease	+ 0.84 (− 1.05, 2.74)	0.38	+ 0.69 (− 1.26, 2.63)	0.49	+ 0.32 (−1.60, 2.24)	0.75
Dialysate measurements						
Calcium						
2.5 mEq/l	reference		reference		reference	
< 2.5 mEq/l	+ 3.18 (− 6.56, 12.91)	0.52	+ 3.03 (− 6.07, 12.14)	0.51	+ 4.00 (− 4.60, 12.60)	0.36
Potassium						
2 mEq/l	reference		reference		reference	
> 2 mEq/l	− 5.44 (− 21.22, 10.35)	0.50	−6.36 (− 21.05, 8.32)	0.40	− 5.58 (− 19.98, 8.82)	0.45
Serum-to-dialysate gradients						
Total calcium, per 1 mEq/l increase in difference	− 11.13 (− 23.59, 1.33)	0.08	−10.81 (− 22.38, 0.76)	0.07	−8.06 (− 18.88, 2.77)	0.15
Corrected calcium, per 1 mEq/l increase in difference	− 9.14 (− 22.83, 4.54)	0.19	− 8.48 (− 21.15, 4.19)	0.2	− 5.61 (− 17.34, 6.12)	0.35

Model 1 includes the main exposure (one of serum, dialysate, or gradient measurements)

Model 2 includes model 1, age, sex, and ethnicity

Model 3 includes model 2, Charlson comorbidity index, non-dialysis systolic blood pressure, left ventricular mass index, and use of antihypertensive medication, renin-angiotensin-aldosterone system blockade, cinacalcet, and QT-prolonging medication

^a^Models with ionized calcium and magnesium also include serum pH

**Fig. 2:**
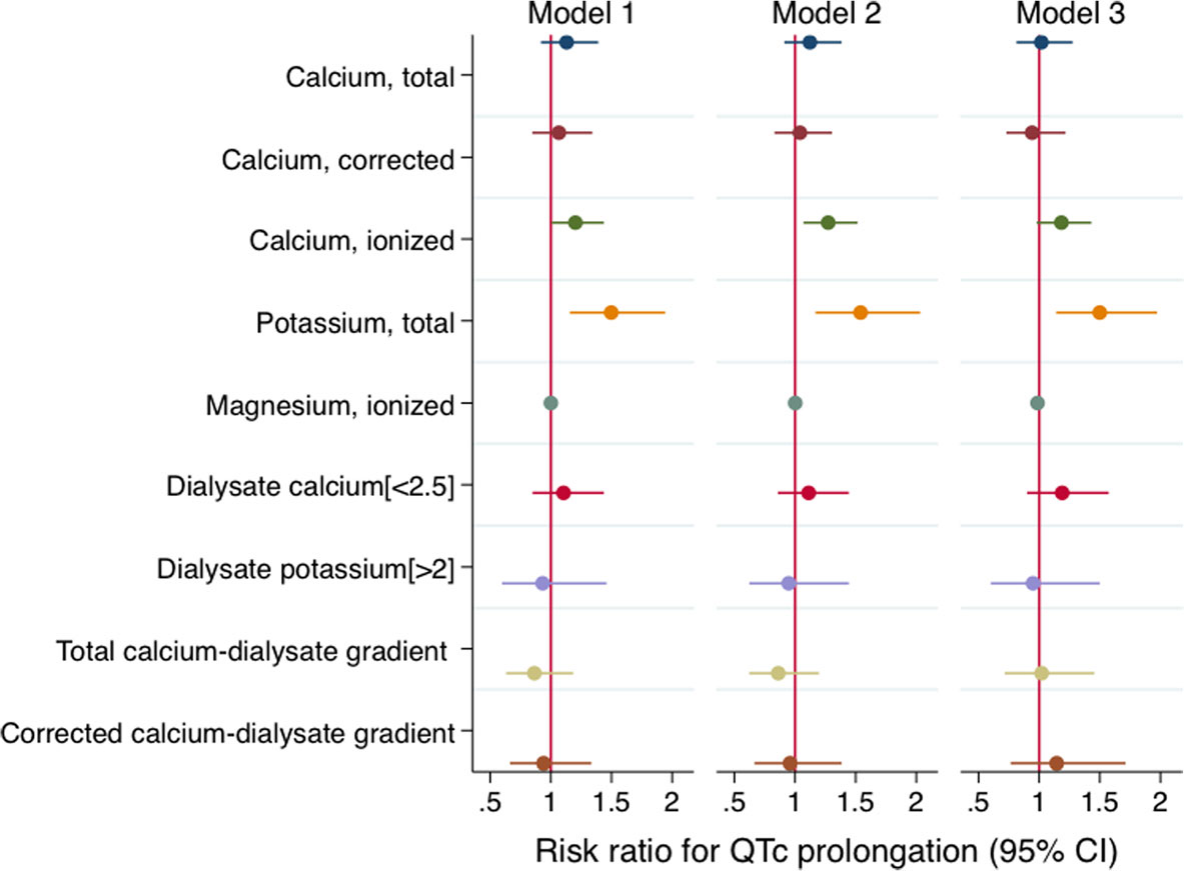
Longitudinal associations of serum, dialysate, and serum-dialysate gradient measures with the risk of QTc prolongation in 146 incident dialysis participants. * Total calcium, per 1 mg/dl decrease; corrected calcium, per 1 mg/dl decrease; ionized calcium, per 0.1 mmol/l decrease; potassium, per 1 mEq/l decrease; magnesium, per 0.1 mEq/l decrease; dialysate calcium [< 2.5] = dialysate calcium < 2.5 mEq/l in reference to 2.5 mEq/l; dialysate potassium [> 2] = dialysate potassium > 2 mEq/l in reference to 2 mEq/l; total calcium-dialysate gradient, per 1 mEq/l increase in difference; corrected calcium-dialysate gradient, per 1 mEq/l increase in difference. Model 1 includes the main exposure (one of serum, dialysate, or gradient measurements). Model 2 includes model 1, age, sex, and ethnicity. Model 3 includes model 2, Charlson comorbidity index, non-dialysis systolic blood pressure, left ventricular mass index, and use of antihypertensive medication, renin-angiotensin-aldosterone system blockade, cinacalcet, and QT-prolonging medication. Models with ionized calcium and magnesium also include serum pH

Lower ionized calcium was associated with a higher risk of QTc prolongation but the association was attenuated in the final model (Fig. 2, Additional file 1: Table S4). Lower serum potassium levels were consistently associated with a higher risk of QTc prolongation. Using restricted cubic splines to examine the predicted probability of QTc prolongation by various measures of calcium and potassium demonstrated that overall, the risk of QTc prolongation increased with lower levels of calcium measures and potassium, but the risk of QTc prolongation was slightly lower in the lowest end of ionized calcium and potassium, and lower around the normal values of calcium and potassium: we observed a drop in the risk around 8.9 mg/dl for serum corrected calcium, 1.18 mmol/l for ionized calcium, and 4.5 mEq/l for serum potassium (Additional file 1: Figure S1). The association of total calcium and risk of QTc prolongation was relatively linear. There was no significant relationship found with QTc interval or QTc prolongation for serum magnesium, dialysate measurements, and serum-to-dialysate gradients over follow-up.

When total serum calcium and potassium measures were simultaneously included in models, lower levels of serum potassium were associated with a longer QTc interval but total serum calcium was not significantly associated with QTc interval. Ionized calcium and serum potassium were both significantly associated with longer QTc interval in the same model (Table 4). Inclusion of with magnesium in the model did not modify the relationship and ionized calcium and serum potassium remained significantly associated with a longer QTc interval. Similar associations were observed with QTc prolongation as the outcome (Additional file 1: Table S5). There was no significant statistical interaction between calcium, potassium, and magnesium measures in their associations with QTc measures.

**Table 4:** Longitudinal associations of serum calcium and ionized calcium with QTc interval in 146 incident dialysis patients after simultaneously adjusting for serum potassium, and the interaction between each serum calcium measure and serum potassium

Variables	Multivariable*		Interaction
	QTc difference (95% CI), ms	P	P**
Serum calcium and potassium in one model			
Total calcium, per 1 SD decrease	+ 0.12 (−0.008, 0.25)	0.07	0.29
Potassium, per 1 SD decrease	+ 0.14 (0.04, 0.23)	0.004	
Ionized calcium and potassium in one model			
Ionized calcium, per 1 SD decrease	+ 0.21 (0.10, 0.32)	< 0.001	0.18
Potassium, per 1 SD decrease	+ 0.13 (0.04, 0.22)	0.003	
Serum calcium and magnesium in one model			
Serum calcium, per 1 SD decrease	+ 0.12 (−0.02, 0.25)	0.08	0.19
Magnesium, per 1 SD decrease	−0.02 (− 0.15, 0.12)	0.82	
Ionized calcium and magnesium in one model			
Ionized calcium, per 1 SD decrease	+ 0.21 (0.10, 0.31)	< 0.001	0.87
Magnesium, per 1 SD decrease	+ 0.01 (−0.13, 0.14)	0.91	
Serum potassium and magnesium in one model			
Serum potassium, per 1 SD decrease	+ 0.13 (0.04, 0.23)	0.006	0.84
Magnesium, per 1 SD decrease	−0.02 (−0.15, 0.12)	0.79	

*Includes main exposures (calcium, potassium, or magnesium), age, sex, ethnicity, Charlson comorbidity index, non-dialysis systolic blood pressure, left ventricular mass index, and use of beta-blocker, renin-angiotensin-aldosterone system blockade, cinacalcet, and QT-prolonging medication. Model with ionized calcium also includes serum pH

**Interaction between calcium and potassium tested in a separate model

## Discussion

Among incident hemodialysis participants, prolonged QTc is very common between dialysis sessions and persists over time. Electrolytes are critical for normal pacing of the heart and low ionized calcium and potassium levels were consistently associated with abnormally prolonged QTc. In contrast, magnesium levels, dialysate, and serum-dialysate gradient for calcium concentrations were not independently associated with prolonged QTc. The results suggest that closer monitoring of lower levels of serum ionized calcium and serum potassium may be important in assessing arrhythmic risk in in-center hemodialysis patients.

Abnormally prolonged QT interval has been associated with adverse cardiovascular outcomes in past studies among the general and CKD population. In the general population, longer QT intervals were predictive of all-cause mortality and cardiovascular mortality in large cohorts over 3 to 30 years follow-up periods, but it is not consistent in all cohorts [20–27]. In the chronic kidney disease population, several studies including the Cardiovascular Health Study (CHS) found that among individuals with CKD or patients receiving hemodialysis or peritoneal dialysis, longer QT interval was associated with an increased risk of death, heart failure, coronary heart disease, and sudden death [28–30]. While QT intervals were also strongly associated with cardiovascular risk factors in these studies, prolonged QT interval remained an independent risk factor for adverse outcomes even after adjusting for traditional risk factors. Prolonged QT intervals may play an important role in risk stratifying individuals with a high risk of cardiovascular mortality.

Electrocardiographic changes, such as QTc prolongation, are common in those on hemodialysis and represent a potential common pathway leading to arrhythmias and potentially sudden cardiac death [4, 6, 31, 32]. We compared multiple measures of serum, ionized, and dialysate electrolytes (calcium, potassium, magnesium) with QTc prolongation, a well-recognized arrhythmogenic risk factor, cross-sectionally and longitudinally, and found that potentially modifiable factors, lower ionized calcium and potassium, consistently and independently predicts QTc prolongation, even after adjusting for use of QT prolonging medications.

Only 5% of the participants were on cinacalcet and thus it is unlikely that this contributed to lower calcium levels and high burden of prolonged QTc interval in our population. Although one third of the participants were receiving a QT prolonging medication, most of which was furosemide, this did not significantly modify the association between calcium and QTc measures. Studies such as the HEMO and DOPPS, which include predominantly prevalent dialysis patients have reported an increased risk of mortality in calcium levels less than 8.5–9.0 mg/dl [5, 33, 34]. We demonstrate that although the associations of serum calcium with QTc prolongation were not statistically significant, lower levels of ionized calcium were associated with increased risk of QTc prolongation.

Our findings also suggest that low predialysis serum potassium is associated with prolonged QTc at baseline and longitudinally, supporting recent studies demonstrating a significant association between serum potassium level and sudden cardiac event or mortality in dialysis patients [12, 35, 36]. Serum potassium has been shown to increase the risk of sudden cardiac arrest and all-cause mortality in dialysis patients with serum potassium levels less than 5.1–5.3 mEq/l or ≤ 4.0 mmol/l. [12, 35, 36] Similarly, we demonstrate that in incident in-center hemodialysis patients, lower levels of serum potassium are associated with longer QTc interval and may consequently contribute to developing other arrhythmias. A recent study of chronic kidney disease participants demonstrated that hyperkalemia (> 5 mEq/l) rather than hypokalemia (< 3.5 mEq/l) was associated with a higher risk of sudden cardiac event [37]. Due to our limited sample size, we could not asses this U-shaped relationship with potassium.

Currently, the use of total versus corrected or ionized calcium is debated when considering routine screening in bundled lab testing and also treating targets according to the KDIGO clinical practice guidelines [38]. Routine measurement of serum ionized calcium is not cost-effective and the use of albumin-corrected calcium remains less specific and non-superior compared to other measures of serum calcium [38, 39]. Our study suggests that ionized calcium is a more consistent and stronger predictor of longer QT interval and QT prolongation, independent of serum potassium levels and other traditional risk factors. As 30% of our study population were found to have QT prolongation at follow-up, most of whom had persistent QT prolongation, closer attention to serum ionized calcium or potassium levels may be important to help mitigate risk of arrhythmias over time. This may be critical especially in dialysis, as the ionized calcium level is the set point for physiological actions including electrophysiology and muscle contraction, and may be more valuable in diagnosis hyperparathyroidism [40].

In recent studies and clinical guidelines, most concerns regarding serum calcium point to potential calcium loading and the risk of vascular calcification [11, 38, 41]. Vascular calcification is common in ESRD [42], and both hypercalcemia and vascular calcification are associated with adverse cardiac events and mortality [43–45]. The 2017 Kidney Disease: Improving Global Outcomes (KDIGO) guidelines weakly suggests that hypercalcemia should be avoided [10]. Although our findings do not show a significant association with serum calcium and prolonged QTc, we show that low levels of ionized calcium is a strong predictor of QTc prolongation, a known arrhythmogenic risk factor, suggesting that methods to monitor and avoid low ionized calcium levels may reduce the risk. Given the concerns for the potential contribution of higher serum calcium levels to vascular calcification, the optimal strategy for maintaining calcium balance in dialysis definitely requires further prospective studies.

Interestingly, our study demonstrates a lack of association between dialysate or serum-dialysate gradient measures and QTc prolongation, even though a few studies have demonstrated significant associations with cardiovascular disease [11]. A case-control study of 502 cardiac arrest cases and 1632 matched controls reported that a dialysate potassium concentration < 2 mEq/l was associated with higher odds of cardiac arrest [12]. We were unable to examine low dialysate potassium concentrations as only 2% of our participants received < 2 mEq/l. In contrast, results from the EVOLVE trial reported no association between baseline dialysate calcium or serum-dialysate calcium gradient with sudden cardiac death [46]. Our results are similar to findings from the EVOLVE trial in that although the direction of the dialysate calcium estimates suggest that lower dialysate calcium concentrations may predict higher arrhythmic risk, the association was not statistically significant. This is most likely due to a much smaller sample size in each category of dialysate calcium. Our study does differ from previous dialysis studies as we examined QTc intervals on interdialytic days and examined electrolytes using multiple predialysis and interdialytic measurements over follow-up.

A limitation of our study is that a smaller proportion of participants completed follow-up cardiovascular evaluations at 1 year; however, the ECG measurements and serum and dialysate electrolytes did not differ by follow-up status. Therefore, while the follow-up cohort may be underpowered, it is less likely that it is biased. Another limitation is the different timing of electrolyte measurements that may potentially influence the observed associations as ionized calcium was done on the same day as the QTc interval measurement and total calcium was performed on routine monthly dialysis testing. Future studies are needed to carefully examine how the timing of measurement can affect the relationship between electrolytes and QT interval. Despite these limitations, important strengths need to be acknowledged. This is a large cohort of incident in-center hemodialysis patients with standardized cardiovascular and clinical characterization and also a large minority population often underrepresented in studies; therefore, findings from this study may be generalizable to these populations.

## Conclusions

Among incident hemodialysis patients, prolonged QTc is extremely common and serum ionized calcium and potassium are inversely associated with QTc interval and the risk of QTc prolongation over time. Dialysate or serum-to-dialysate gradient measures were not associated with prolonged QTc. Our findings suggest that monitoring of serum ionized calcium and potassium may mitigate prolonged QTc and its potential risk for arrhythmias and sudden cardiac death.

## Additional file

Additional file 1: **Table S1.** Tabulation of QT prolonging medications at the first study visit. **Table S2.** Baseline associations of serum and dialysate electrolytes with the risk of QTc prolongation in 330 incident dialysis patients. **Table S3.** Comparison of baseline characteristics between participants who completed a follow-up visit and participants who did not complete a follow-up visit among those who survived to 1 year after the first study clinic visit. **Table S4.** Longitudinal associations of serum and dialysate electrolytes with the risk of QTc prolongation in 146 participants. **Figure S1.** Predicted probabilities of QTc prolongation by (A) serum total calcium, (B) corrected calcium, (C) ionized calcium, and (D) potassium using restricted cubic splines from longitudinal analysis of serum and dialysate electrolytes with the risk of QTc prolongation in 146 participants. **Figure S2.** Longitudinal associations of serum calcium and ionized calcium with the risk of QTc prolongation in 146 participants after simultaneously adjusting for serum potassium, and the interaction between each serum calcium measure and serum potassium. (DOCX 433 kb)
