# Supplementary material for: Associations of serum and dialysate electrolytes with QT interval and prolongation in incident hemodialysis: the Predictors of Arrhythmic and Cardiovascular Risk in End-Stage Renal Disease (PACE) study
Source: BMC Nephrol. 2019 Apr 18;20:133. doi: 10.1186/s12882-019-1282-5 (PMC6474045; doi:10.1186/s12882-019-1282-5)
Supplement: Supplementary file 1 — Table S1. Tabulation of QT prolonging medications at the first study visit. Table S2. Baseline associations of serum and dialysate electrolytes with the risk of QTc prolongation in 330 incident dialysis patients. Table S3. Comparison of baseline characteristics between participants who completed a follow-up visit and participants who did not complete a follow-up visit among those who survived to 1 year after the first study clinic visit. Table S4. Longitudinal associations of serum and dialysate electrolytes with the risk of QTc prolongation in 146 participants. Figure S1. Predicted probabilities of QTc prolongation by (A) serum total calcium, (B) corrected calcium, (C) ionized calcium, and (D) potassium using restricted cubic splines from longitudinal analysis of serum and dialysate electrolytes with the risk of QTc prolongation in 146 participants. Figure S2. Longitudinal associations of serum calcium and ionized calcium with the risk of QTc prolongation in 146 participants after simultaneously adjusting for serum potassium, and the interaction between each serum calcium measure and serum potassium. (DOCX 433 kb) [file 12882_2019_1282_MOESM1_ESM.docx]

**Table S1:** Tabulation of QT prolonging medications at the first study visit

| **QT prolonging medication* (Generic, Brand name)** | **N** | **%** |
| --- | --- | --- |
| Alfuzosin, Uroxatral | 1 | 0.58 |
| Amiodarone, Cordarone | 1 | 0.58 |
| Aripiprazole, Abilify | 1 | 0.58 |
| Atazanavir, Reyataz | 7 | 4.05 |
| Azithromycin, Zithromax | 4 | 2.31 |
| Ciprofloxacin, Cipro | 3 | 1.73 |
| Citalopram, Celexa | 1 | 0.58 |
| Diphenhydramine, Benadryl | 6 | 3.47 |
| Doxepin, Sinequan | 1 | 0.58 |
| Erythromycin, E.E.S. | 1 | 0.58 |
| Escitalopram, Cipralex | 7 | 4.05 |
| Famotidine, Pepcid | 3 | 1.73 |
| Fluoxetine, Prozac | 2 | 1.16 |
| Furosemide (Frusemide), Lasix | 52 | 30.06 |
| Haloperidol, Haldol | 1 | 0.58 |
| Iloperidone, Fanapt | 1 | 0.58 |
| Indapamide, Lozol | 1 | 0.58 |
| Isradipine, Dynacirc | 1 | 0.58 |
| Lansoprazole/Amoxicillin/Clarithromycin | 2 | 1.16 |
| Lopinavir/Ritonavir, Kaletra | 2 | 1.16 |
| Methadone, Dolophine | 2 | 1.16 |
| Metronidazole, Flagyl | 2 | 1.16 |
| Mirtazapine, Remeron | 4 | 2.31 |
| Moxifloxacin, Avelox | 1 | 0.58 |
| Nortriptyline, Pamelor | 2 | 1.16 |
| Olanzapine, Zyprexa | 3 | 1.73 |
| Ondansetron, Zofran | 2 | 1.16 |
| Paliperidone, Invega | 1 | 0.58 |
| Paroxetine, Paxil | 2 | 1.16 |
| Promethazine, Phenergan | 7 | 4.05 |
| Quetiapine, Seroquel | 2 | 1.16 |
| Ranolazine, Ranexa | 1 | 0.58 |
| Risperidone, Risperdal | 1 | 0.58 |
| Ritonavir, Norvir | 11 | 6.36 |
| Sertraline, Zoloft | 9 | 5.2 |
| Solifenacin, Vesicare | 1 | 0.58 |
| Tacrolimus, Prograf | 1 | 0.58 |
| Tolterodine, Detrol | 8 | 4.62 |
| Trazodone, Desyrel | 6 | 3.47 |
| Trimethoprim-Sulfa, Septra | 7 | 4.05 |
| Venlafaxine, Effexor | 1 | 0.58 |
| Voriconazole, VFend | 1 | 0.58 |
| Ziprasidone, Geodon | 1 | 0.58 |
| Total | 173** | 100 |

* Woosley, RL and Romero, KA, www.Crediblemeds.org, QTdrugs List, January 2016, AZCERT, Inc. 1822 Innovation Park Dr., Oro Valley, AZ 85755

** Adds up to more than 118 because some patients received multiple QT prolonging medications

**Table S2:** Baseline associations of serum and dialysate electrolytes with the risk of QTc prolongation in 330 incident dialysis patients

| Variables | **Model 1** | | **Model 2** | | **Model 3** | | |  |
| --- | --- | --- | --- | --- | --- | --- | --- | --- |
|  | **RR (95% CI)** | **P** | **RR (95% CI)** | **P** | **RR (95% CI)** | **P** | | |
| **Serum measurements** |  |  |  |  |  |  | | |
| **Total calcium**, per 1 mg/dl decrease | 1.04 (0.87, 1.25) | 0.65 | 1.04 (0.87, 1.24) | 0.65 | 1.02 (0.85, 1.22) | 0.86 | | |
| **Corrected calcium**, per 1 mg/dl decrease | 1.03 (0.84, 1.26) | 0.78 | 1.02 (0.83, 1.25) | 0.83 | 1.00 (0.81, 1.23) | 0.99 | | |
| **Ionized calcium***, per 0.1 mmol/l decrease | **1.30 (1.12, 1.51)** | **0.001** | **1.33 (1.14, 1.55)** | **<0.001** | **1.31 (1.12, 1.53)** | **0.001** | | |
| **Potassium**, per 1 mEq/l decrease | 1.23 (0.98, 1.53) | 0.07 | **1.25 (1.00, 1.55)** | **0.05** | 1.20 (0.97, 1.49) | 0.09 | | |
| **Magnesium***, per 0.1 mEq/l decrease | 0.99 (0.94, 1.04) | 0.62 | 0.99 (0.94, 1.04) | 0.59 | 0.97 (0.92, 1.02) | 0.26 | | |
| **Dialysate measurements** |  |  |  |  |  |  | | |
| **Calcium** |  |  |  |  |  |  | | |
| **2.5** mEq/l | reference |  | reference |  |  |  | | |
| < **2.5** mEq/l | 0.95 (0.76, 1.20) | 0.68 | 0.96 (0.77, 1.21) | 0.76 | 1.01 (0.80, 1.27) | 0.94 | | |
| **Potassium** |  |  |  |  |  |  | | |
| **2** mEq/l | reference |  | reference |  |  |  | | |
| **> 2** mEq/l | 1.05 (0.76, 1.44) | 0.77 | 1.05 (0.77, 1.42) | 0.77 | 1.02 (0.77, 1.37) | 0.87 | | |
| **Serum-to-dialysate gradients** |  |  |  |  |  |  | | |
| **Total calcium**, per 1 mEq/l increase in difference | 0.91 (0.69, 1.19) | 0.47 | 0.90 (0.69, 1.19) | 0.47 | 0.96 (0.72, 1.28) | 0.80 | | |
| **Corrected calcium**, per 1 mEq/l increase in difference | 0.92 (0.68, 1.23) | 0.55 | 0.92 (0.69, 1.24) | 0.58 | 0.98 (0.72, 1.34) | 0.92 | | |
| RR = risk ratio  Model 1 includes the main exposure (one of serum, dialysate, or gradient measurements)  Model 2 includes model 1, age, sex, and ethnicity  Model 3 includes model 2, Charlson comorbidity index, non-dialysis systolic blood pressure, left ventricular mass index, and use of antihypertensive medication, renin-angiotensin-aldosterone system blockade, cinacalcet, and QT-prolonging medication  * Models with ionized calcium and magnesium also include serum pH | | | | | | |  |  |

**Table S3:** Comparison of baseline characteristics between participants who completed a follow-up visit and participants who did not complete a follow-up visit among those who survived to 1 year after the first study clinic visit

| **Variables** | **Mean (± SD), median (IQR), frequency (%)** | | **P** |
| --- | --- | --- | --- |
|  | **Participants without follow-up assessment**  **(n=149)** | **Participants with follow-up assessment**  **(n=146)** |  |
| **Demographic factors** |  |  |  |
| Age, years | 53.6 (13.8) | 55.2 (13.6) | 0.31 |
| Male sex | 94 (63.1%) | 90 (61.6%) | 0.80 |
| African American ethnicity | 104 (69.8%) | 117 (80.1%) | 0.04 |
| **Physical information** |  |  |  |
| Body mass index, kg/m^2^ | 28.0 (23.9, 33.3) | 27.9 (24.3, 33.8) | 0.46 |
| Non-dialysis systolic blood pressure, mmHg | 137.5 (24.5) | 138.0 (26.6) | 0.86 |
| Non-dialysis diastolic blood pressure, mmHg | 74.7 (15.6) | 75.5 (14.4) | 0.63 |
| Non-dialysis study visit pulse pressure, mmHg | 62.8 (17.4) | 62.5 (19.5) | 0.89 |
| **Comorbidities** |  |  |  |
| Hypertension | 149 (100.0%) | 146 (100.0%) | n/a |
| Hypercholesterolemia | 103 (69.1%) | 103 (70.5%) | 0.79 |
| Diabetes | 80 (53.7%) | 82 (56.2%) | 0.67 |
| Coronary artery disease | 56 (37.6%) | 48 (32.9%) | 0.40 |
| Charlson comorbidity index | 5 (3, 6) | 5 (4, 6) | 0.41 |
| **Medications** |  |  |  |
| Total number of antihypertensive medications | 2.7 (1.4) | 2.7 (1.3) | 0.91 |
| Beta-blocker | 92 (68.7%) | 100 (73.5%) | 0.38 |
| RAAS^†^ blockade | 49 (36.6%) | 63 (46.3%) | 0.10 |
| Calcium channel blockers | 82 (61.2%) | 87 (64.0%) | 0.64 |
| Alpha blockers | 18 (13.4%) | 13 (9.6%) | 0.32 |
| Vasodilators | 46 (34.3%) | 49 (36.0%) | 0.77 |
| Diuretic | 32 (23.9%) | 29 (21.3%) | 0.62 |
| Cinacalcet | 7 (5.2%) | 6 (4.4%) | 0.76 |
| QT prolonging medications | 46 (30.9%) | 59 (40.4%) | 0.09 |
| **Cardiac parameters** |  |  |  |
| Left ventricular mass index, g/m^2.7^ | 60.5 (49.6, 81.6) | 61.7 (50.1, 79.5) | 0.66 |
| Left ventricular ejection fraction, % | 65.7 (12.2) | 66.3 (12.1) | 0.66 |
| **Laboratory measurements** |  |  |  |
| Hemoglobin*, g/dl | 10.7 (1.2) | 10.9 (1.3) | 0.09 |
| pH^‡^ | 7.34 (0.04) | 7.34 (0.04) | 0.08 |
| Total calcium*, mg/dl | 8.8 (0.5) | 8.8 (0.7) | 0.39 |
| Corrected calcium*, mg/dl | 9.0 (0.5) | 9.1 (0.6) | 0.05 |
| Ionized calcium^‡^, mmol/l | 1.15 (0.06) | 1.14 (0.08) | 0.71 |
| Total potassium*, mEq/l | 4.4 (0.5) | 4.4 (0.5) | 0.64 |
| Magnesium^‡^, mEq/l | 1.77 (0.21) | 1.75 (0.27) | 0.46 |
| **Dialysate measurements** |  |  |  |
| Calcium^§^, mEq/l |  |  |  |
| 2 | 60 (40.3%) | 62 (42.5%) | 0.78 |
| 2.25 | 5 (3.4%) | 8 (5.5%) |  |
| 2.5 | 83 (55.7%) | 75 (51.4%) |  |
| 3 | 1 (0.7%) | 1 (0.7%) |  |
| Potassium^§^, mEq/l |  |  |  |
| 1 | 0 (0.0%) | 5 (3.4%) | 0.07 |
| 2 | 127 (85.2%) | 119 (81.5%) |  |
| 3 | 22 (14.8%) | 22 (15.1%) |  |
| **Serum-dialysate measurements** |  |  |  |
| Total calcium*, mEq/l | 2.1 (0.4) | 2.1 (0.4) | 0.32 |
| Corrected calcium*, mEq/l | 2.2 (0.4) | 2.3 (0.4) | 0.06 |
| **ECG measurements** |  |  |  |
| QT interval^‡^, ms | 435.5 (58.2) | 436.1 (53.1) | 0.92 |
| Corrected QT interval^‡^, ms | 453.1 (48.4) | 456.4 (44.7) | 0.54 |
| QT prolongation^‡^ | 68 (45.6%) | 69 (47.3%) | 0.78 |
| ^†^Renin-angiotensin-aldosterone system blockade includes angiotensin-converting-enzyme inhibitor and angiotensin II receptor blocker | | | |
| *3-month average predialysis measurements  ^‡^Non-dialysis (interdialytic) measurements  ^§^Measurement closest to the study clinic visit | | | |

**Table S4:** Longitudinal associations of serum and dialysate electrolytes with the risk of QTc prolongation in 146 participants

| Variables | **Model 1** | | **Model 2** | | **Model 3** | | |  |
| --- | --- | --- | --- | --- | --- | --- | --- | --- |
|  | **RR (95% CI)** | **P** | **RR (95% CI)** | **P** | **RR (95% CI)** | **P** | | |
| **Serum measurements** |  |  |  |  |  |  | | |
| **Total calcium**, per 1 mg/dl decrease | 1.13 (0.92, 1.39) | 0.25 | 1.12 (0.91, 1.38) | 0.27 | 1.02 (0.81, 1.28) | 0.88 | | |
| **Corrected calcium**, per 1 mg/dl decrease | 1.07 (0.85, 1.34) | 0.58 | 1.04 (0.83, 1.30) | 0.73 | 0.94 (0.73, 1.22) | 0.65 | | |
| **Ionized calcium***, per 0.1 mmol/l decrease | **1.20 (1.01, 1.44)** | **0.04** | **1.21 (1.01, 1.46)** | **0.04** | 1.18 (0.98, 1.43) | 0.08 | | |
| **Potassium**, per 1 mEq/L decrease | **1.50 (1.16, 1.94)** | **0.002** | **1.54 (1.17, 2.03)** | **0.002** | **1.50 (1.14, 1.97)** | **0.004** | | |
| **Magnesium***, per 0.1 mEq/l decrease | 1.00 (0.95, 1.06) | 0.98 | 1.00 (0.95, 1.05) | 0.95 | 0.99 (0.93, 1.04) | 0.65 | | |
| **Dialysate measurements** |  |  |  |  |  |  | | |
| **Calcium** |  |  |  |  |  |  | | |
| **2.5** mEq/l | reference |  | reference |  | reference |  | | |
| < **2.5** mEq/l | 1.11 (0.85, 1.44) | 0.46 | 1.11 (0.86, 1.44) | 0.42 | 1.19 (0.90, 1.57) | 0.22 | | |
| **Potassium** |  |  |  |  |  |  | | |
| **2** mEq/l | reference |  | reference |  | reference |  | | |
| **> 2** mEq/l | 0.93 (0.60, 1.46) | 0.76 | 0.95 (0.62, 1.44) | 0.81 | 0.95 (0.60, 1.50) | 0.83 | | |
| **Serum-to-dialysate gradients** |  |  |  |  |  |  | | |
| **Total calcium**, per 1 mEq/l increase in difference | 0.86 (0.63, 1.18) | 0.36 | 0.86 (0.62, 1.20) | 0.38 | 1.02 (0.72, 1.45) | 0.91 | | |
| **Corrected calcium**, per 1 mEq/l increase in difference | 0.94 (0.66, 1.33) | 0.73 | 0.96 (0.67, 1.38) | 0.83 | 1.14 (0.77, 1.71) | 0.51 | | |
| RR = risk ratio  Model 1 includes the main exposure (one of serum, dialysate, or gradient measurements)  Model 2 includes model 1, age, sex, and ethnicity  Model 3 includes model 2, Charlson comorbidity index, non-dialysis systolic blood pressure, left ventricular mass index, and use of antihypertensive medication, renin-angiotensin-aldosterone system blockade, cinacalcet, and QT-prolonging medication  * Models with ionized calcium and magnesium also include serum pH | | | | | | |  |  |

**Figure S1:** Predicted probabilities of QTc prolongation by (A) serum total calcium, (B) corrected calcium, (C) ionized calcium, and (D) potassium using restricted cubic splines from longitudinal analysis of serum and dialysate electrolytes with the risk of QTc prolongation in 146 participants

Models include the main exposure (either calcium or potassium), age, sex, ethnicity, Charlson comorbidity index, non-dialysis systolic blood pressure, left ventricular mass index, and use of antihypertensive medication, renin-angiotensin-aldosterone system blockade, cinacalcet, and QT-prolonging medication. Model with ionized calcium additionally includes serum pH.

**Table S5:** Longitudinal associations of serum calcium and ionized calcium with the risk of QTc prolongation in 146 participants after simultaneously adjusting for serum potassium, and the interaction between each serum calcium measure and serum potassium

| **Variables** | **Multivariable^*^** | | **Interaction** |
| --- | --- | --- | --- |
|  | **RR (95% CI)** | **P** | **P**** |
| **Serum calcium and potassium in one model** |  |  |  |
| **Total calcium**, per 1 SD decrease | 1.01 (0.86, 1.18) | 0.90 | 0.64 |
| **Potassium**, per 1 SD decrease | **1.26 (1.08, 1.47)** | **0.004** |  |
| **Ionized calcium and potassium in one model** |  |  |  |
| **Ionized calcium**, per 1 SD decrease | **1.21 (1.04, 1.40)** | **0.01** | 0.29 |
| **Potassium**, per 1 SD decrease | **1.26 (1.09, 1.46)** | **0.002** |  |
| **Serum calcium and magnesium in one model** |  |  |  |
| **Serum calcium**, per 1 SD decrease | 1.02 (0.87, 0.18) | 0.85 | 0.64 |
| **Magnesium**, per 1 SD decrease | 1.02 (0.87, 1.19) | 0.84 |  |
| **Ionized calcium and magnesium in one model** |  |  |  |
| **Ionized calcium**, per 1 SD decrease | **1.21 (1.05, 1.39)** | **0.01** | 0.97 |
| **Magnesium**, per 1 SD decrease | 1.06 (0.90, 1.25) | 0.51 |  |
| **Serum potassium and magnesium in one model** |  |  |  |
| **Serum potassium**, per 1 SD decrease | **1.26 (1.08, 1.47)** | **0.004** | 0.81 |
| **Magnesium**, per 1 SD decrease | 1.03 (0.88, 1.20) | 0.75 |  |
| * Includes main exposures (calcium, potassium, or magnesium), age, sex, ethnicity, Charlson comorbidity index, non-dialysis systolic blood pressure, left ventricular mass index, and use of beta-blocker, renin-angiotensin-aldosterone system blockade, cinacalcet, and QT-prolonging medication. Model with ionized calcium also includes serum pH.  ** Interaction between calcium and potassium tested in a separate model | | | |
